# Evaluation of 11 Scoring Functions Performance on Matrix Metalloproteinases

**DOI:** 10.1155/2014/162150

**Published:** 2014-12-25

**Authors:** Jamal Shamsara

**Affiliations:** Pharmaceutical Research Center, Mashhad University of Medical Sciences, Mashhad 91775-1365, Iran

## Abstract

Matrix metalloproteinases (MMPs) have distinctive roles in various physiological and pathological processes such as inflammatory diseases and cancer. This study explored the performance of eleven scoring functions (D-Score, G-Score, ChemScore, F-Score, PMF-Score, PoseScore, RankScore, DSX, and X-Score and scoring functions of AutoDock4.1 and AutoDockVina). Their performance was judged by calculation of their correlations to experimental binding affinities of 3D ligand-enzyme complexes of MMP family. Furthermore, they were evaluated for their ability in reranking virtual screening study results performed on a member of MMP family (MMP-12). Enrichment factor at different levels and receiver operating characteristics (ROC) curves were used to assess their performance. Finally, we have developed a PCA model from the best functions. Of the scoring functions evaluated, F-Score, DSX, and ChemScore were the best overall performers in prediction of MMPs-inhibitors binding affinities while ChemScore, Autodock, and DSX had the best discriminative power in virtual screening against the MMP-12 target. Consensus scorings did not show statistically significant superiority over the other scorings methods in correlation study while PCA model which consists of ChemScore, Autodock, and DSX improved overall enrichment. Outcome of this study could be useful for the setting up of a suitable scoring protocol, resulting in enrichment of MMPs inhibitors.

## 1. Introduction

Matrix metalloproteinases (MMPs) are zinc-dependent endopeptidases that play a central role in various physiological processes and pathological conditions including cancer and inflammatory diseases. One of the main problems for developing a new class of drugs as MMP inhibitors is the issue of selectivity. This family shares a very similar active site that makes traditional chemical approach for developing of selective inhibitors time-consuming. In this case the computational approaches including molecular docking can help the medicinal chemistry [1, 2].

As reliability of different scoring functions is very target-dependent [3], in this study we aimed to evaluate some available scoring functions in scoring of MMPs-ligands interactions. Reliability of molecular docking depends on how the geometry of ligands will be predicted and how the different pose of a ligand and interaction of different ligands with receptor will be ranked [4]. The former has been investigated on a set of 40 MMPs complexes [5]. In our paper we focused on successfully ranking the interaction of different ligands with MMPs. Scoring functions are used to estimate the binding affinity of a compound for a receptor in a reasonable time. These scoring functions can fall into three categories [6, 7]: (1) empirical scoring functions, including X-Score [8], F-Score [9, 10], and ChemScore [11], (2) knowledge-based potentials, including DSX [12] and PMF-Score [13], and (3) force-field based approaches, including D-Score [14] and G-Score [15]. Knowledge-based scoring functions observe interatomic contact frequencies and/or distances in a large database of protein-ligand complexes 3D structures. The observed frequency distributions of favorable and unfavorable molecular interactions are converted to potentials of mean force or knowledge-based potentials. The two other mentioned categories contain scoring methods based on physical interaction terms. These methods try to estimate the change in free energy upon ligand binding via decomposition of free energy into a sum of individual contributions. The first class of scoring functions within this group (force-field based) directly derives the interaction terms from physicochemical theory and does not fit them to experimental data. The other class (empirical based) tries to find linear statistical relationship between the binding affinity and a number of ligand binding terms in a training set of ligand-protein complexes 3D structures with associated binding affinity data [4].

Some proposed consensus docking [16, 17] and consensus rescoring [18] protocols are available. The two consensus scoring methods so-called rank-by-number and rank-by-rank that had shown promising results [18] were also tested in this study. In addition, we suggested another method, principal component analysis (PCA), for performing a combination of multiple scoring functions to rescore and rerank the compounds after virtual screening on MMP-12 target.

The work reported here seeks to address two questions. (1) How can different scoring functions predict the experimental binding affinities for MMPs-inhibitor complexes? (2) Do the well-performed scoring functions have also reasonable performance in an enrichment study on a member of MMPs family (MMP-12)?

## 2. Methods

### 2.1. Preparation of Protein Test Set for Rescoring Study

The test set consisted of 100 MMPs-ligand complex structures formed of 10 human MMPs types. We excluded the structures with conflictive reported binding affinities. The 3D structures were taken from PDB (Protein Data Bank) and then underwent some refinements. Firstly, water and other cocrystalized molecules were removed from the retrieved PDB files. Then, the protein and corresponding ligand (inhibitor) were extracted to separate PDB files. The file formats changed to mol2 as it was a necessary step for some subsequent analysis. The hydrogens were added to both protein and ligand molecules. All of the selected PDB structures had experimentally determined Ki, Kd, or IC50. The logarithm of Ki, Kd, or IC50 was employed as experimental binding affinity in our study. The detailed structural information for each is presented in Supplementary Material available online at http://dx.doi.org/10.1155/2014/162150. For analysis, the pAffinity was employed as dependent variable instead of binding affinity which was defined as below:

 pAffinity = −Log (Ki, Kd or IC50) (*μ*M).

Metal ion (catalytic zinc ion) was saved as a part of the macromolecule. The Gasteiger partial charge was assigned for ligands. All of the above procedures were done using the PyMOL (http://www.pymol.org/, The PyMOL Molecular Graphics System, version 1.2r3pre, Schrödinger, LLC) and Open Babel Package (version 2.3.1 http://openbabel.org/) [19].

### 2.2. Scoring Functions

Various scoring functions have been evaluated in this study. 11 scoring functions including the five SYBYL built-in scoring functions (D-Score [14], G-Score [15, 20], ChemScore [11], F-Score [9, 10], and PMF_Score [13]), two web based scoring functions (PoseScore [21] and RankScore [21]), two standalone scoring functions (DSX [12] and X-Score [8]), and scoring functions of AutoDock4.1 [22, 23] and AutoDock Vina [24] were employed in this study. Furthermore, two consensus scorings were applied on the set. All of the 11 scoring functions were used to compute binding scores for ligand-protein interactions. Some of the scoring functions were not able to compute reasonable binding score for all of the complexes. It has been discussed earlier that such an incompatibility may be raised by the fact that in some cases there are clashes between protein and ligand molecules [25]. However, we did not penalize those scoring functions in our study. The pairwise deletion strategy was used to deal with missing data.

We employed previously defined consensus scoring (rank-by-number and rank-by-rank methods [18]) to summarize the results of multiple scoring functions. In rank-by-rank method, predicted individual rank was calculated as an average of ranks predicted by all the scoring functions. Rank-by-number consensus score is an average of the Z-scaled scores calculated by each of the individual scoring functions. Individual Z-scaled scoring function values (ZScore) are computed by

(1) $$\begin{array}{c} \text{Zscore}=\frac{\left(f_{i}-\mu \right)}{S}, \end{array}$$

where *f*_*i*_ is the scoring value of an individual scoring function, *μ* is the mean value, and *S* is the standard deviation of this scoring function for entire set.

Finally, the principal component analysis (PCA) was applied on various set of scores of enrichment study to evaluate the discrimination power of PCA on our evaluated set of compounds. PCA is a powerful tool for different aspects of data evaluation including classification and pattern recognition. It can simplify and reduce the dimensionality of multivariate data set while preserving as much of the relevant information. The principal components (PCs) are linear combinations of the original variables. The first principal component (PC1) has the largest possible variance. The second principal component (PC2) is uncorrelated to the first one, and it accounts for most of the remaining variance. PCA model has been employed in our study for discrimination of actives among decoys in virtual screening results based on obtained scores from various scoring functions. In case of our study the PCA was applied to generate linear combination of different scores and extracted the main variation in the data as PC1 and subsequent rescoring and reranking of virtual screening results based on formulated PC1. The contribution of an individual score to the calculated PC can be described by its loading value.

### 2.3. Preparation of Docking Set for the Retrospective Virtual Screening on MMP-12

The inhibitors molecules of docking set were prepared basically from the MMP-12 inhibitors spreadsheet taken from ChEMBL database [26]. Firstly, inactive and low active molecules (IC50 > 100,000 nM) were removed from the spreadsheet. Cases with incomplete information (unitless activity or inexact IC50 values) and those which did not fully satisfy Lipinski'srule of five were also excluded from the spreadsheet. The edited spreadsheet containing SMILES and bioactivities was imported in Canvas 1.6. Some of the selected inhibitors from the previous step shared similar scaffolds and it could cause biased results. To overcome this potential problem finger prints for every inhibitor were defined by binary fingerprint module. Then, diversity selection tool was applied to select the most 30 diversified molecules from inhibitor set. The 30 inhibitors were visually inspected to have different scaffolds. To generate decoys which physically resemble active set we used the online-tool of DUD-E [27]. It generated 50 decoys for each active molecule. In summary, this tool tries to make decoys with similar physical properties including molecular weight, calculated logP, number of rotatable bonds, and hydrogen bond donors and acceptors for each ligand while it minimizes the 2D topological similarities between generated decoys and corresponding ligand to make them suitable for true negative control role. 3D conformations were generated for actives and decoys and subsequent energy minimization, partial charge assignment, and ionization were performed. These steps were done using LigPrep module in Schrödinger. It uses force field OPL2005 for energy minimization after 2D to 3D conversion of ligands.

### 2.4. Docking and Preparation of the Protein for the Retrospective Virtual Screening on MMP-12

The Glide (Glide, version 5.7, Schrödinger, LLC, New York, NY, 2011) was used for docking studies. As mentioned above a set of inhibitors and decoys was docked in MMP-12 (PDB code: 3F17) active site. For receptor preparation, water molecules were removed, hydrogens were added, and protein structure was minimized using protein preparation wizard [28]. For Glide, two docking runs were conducted: a docking procedure with high*-*throughput virtual screening (HTVS) setting and another one with standard precision (SP) mode. We used default settings. Grid box was centered at cocrystalized ligand and was sized to 14 angstrom. The output files were saved as mol2 format.

### 2.5. Statistical Analysis

The scoring functions were evaluated via calculation of the linear correlation between predicted binding affinity scores and experimentally determined binding affinities. Pearson's correlation coefficient (*R*_*p*_) and Spearman's correlation coefficient (*R*_*s*_) were used for quantitative assessment of scoring functions predictivity. Pearson's correlation coefficient shows the predictivity of scores while Spearman's correlation coefficient indicates the predictive ability of scoring functions to properly rank the ligand-receptor affinities.

To evaluate the performance of the scoring functions in discriminating actives among decoys the scoring functions performance was tested on docked active and decoy compounds. The receiver operating characteristic (ROC) curve and enrichment factor (EF) were applied to determine the performance of each scoring function. The increase in area under the curve (AUC) of ROC curve can be used as an indicator of improvement in discrimination between true ligands from decoys. AUC can have a value between 0 and 1, in which AUC = 0.5 means that the method of interest performed like a random selection in average, while AUC = 1 means the complete discrimination between true and false cases (active and decoys). EF is defined as the fraction of active compounds found divided by the fraction of the screened library:

(2) $$\begin{array}{c} \text{EF}=\left(\frac{{\text{actives}}_{\text{sampled}}}{{\text{actives}}_{\text{total}}}\right)\times \left(\frac{N_{\text{total}}}{N_{\text{sampled}}}\right). \end{array}$$

EF1% and EF2% are shown the ability of a particular scoring method to retrieve true ligands with a high rank among virtual screening results. They could be even more informative than AUC of ROC curve index, as scoring functions with AUC of ROC curve around 0.5 could still have an acceptable performance at early stage of the curve that can be detected using EF1% or EF2%.

All of the statistical test and plotting were done using R (R: a language and environment for statistical computing; R Foundation for Statistical Computing, Vienna, Austria; URL http://www.R-project.org/.) including packages: enrichvs, missMDA, and ROCR [29].

## 3. Results

### 3.1. Correlation of Predicted Scores with Experimental Binding Affinities

**Figure 1:**
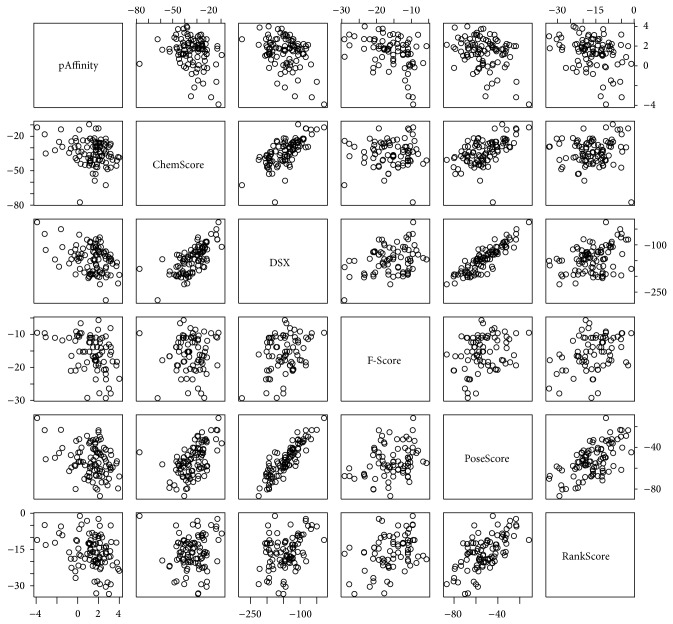
Scatter plot for the best performed scoring functions. Correlation of each scoring function relative to other scoring functions as well as experimental binding affinity (pAffinity) is shown.

The −Log experimental binding affinities (pAffinity) for the selected test set of MMPs-ligand complexes range from −3.9 to 4, spanning about 8 orders of magnitude with a mean value of 1.40 and STD of 1.52 (Supplementary Material). The correlation table of scoring functions (scores from all the 11 scoring functions as well as two consensus scorings) are shown in Supplementary Material.  Table 1 shows the correlation coefficients between different scoring functions and pAffinity.  Table 2 summarized the main results of the scoring functions comparison. The consensus scorings did not improve the prediction more than the best scoring functions. For scoring functions which had a good correlation with experimental results (F-Score, PoseScore, RankScore, DSX, and ChemScore), correlation plots are shown in Figure 1.

### 3.2. ROC Curve Analysis and Enrichment Factor Calculation for the Retrospective Virtual Screening on MMP-12

Based on the fact that PoseScore and RankScore have online based interfaces they were excluded from rescoring assessment.

**Figure 2:**
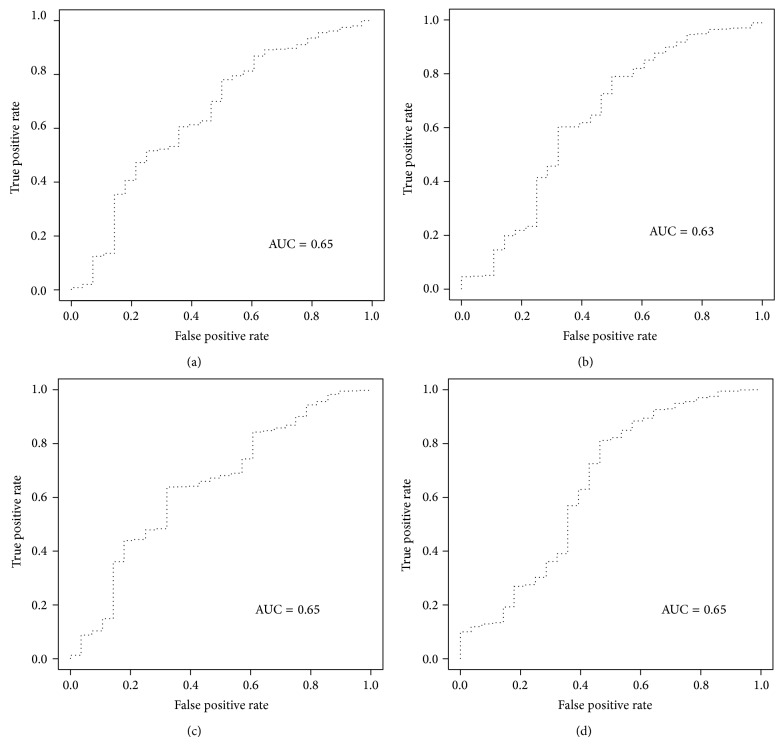
ROC curve of (a) Glide-Score, (b) DSX, (c) Autodock, and (d) ChemScore for Glide (HTS) virtual screening results.

**Figure 3:**
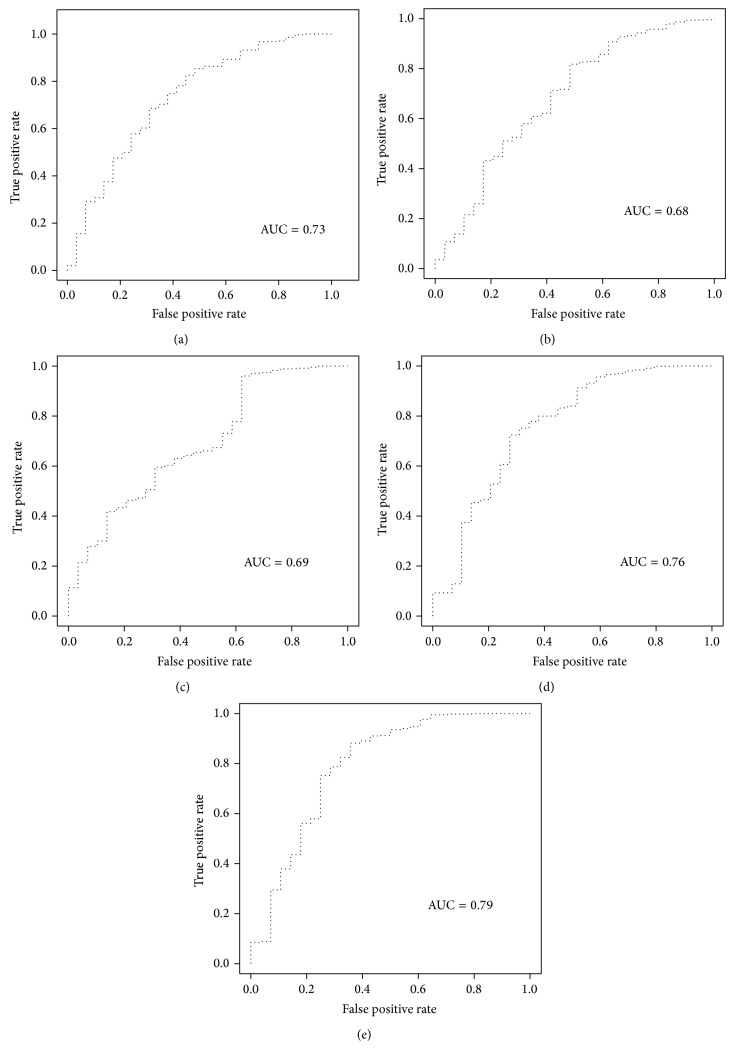
ROC curve of (a) Glide-Score, (b) DSX, (c) Autodock, (d) ChemScore, and (e) PC1 for Glide (SP) virtual screening results.

**Figure 4:**
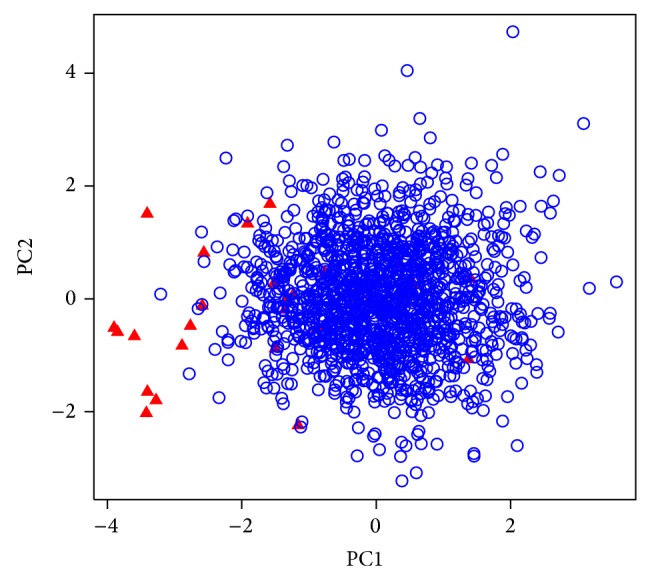
Plot of PC2 against PC1 for Glide virtual screening results (SP). ▲: actives; ○: decoys.

ROC curve plots specificity against sensitivity at different cutoff values (in this case, different scores). The enrichment ability of scoring functions was assessed on a set of docked compounds including known inhibitors and decoys.  Table 3 demonstrated the obtained EFs at different level for various scoring function on docked poses with either Glide standard precision or Glide HTVS protocols. In addition to Glide-native scoring function, ChemScore, Autodock, and DSX showed better performance than other tested functions in both rescoring jobs. Figures 2 and 3 show representative ROC plots for scoring functions with the best performances among scoring programs evaluated in the enrichment study. The calculated areas under the receiver-operating characteristic curves values for each scoring program are given in Table 3. PC1 obtained from performed PCA on Autodock, DSX, and ChemScore scores led to the best EF1% and AUC for SP docking runs. Principle component 2 (PC2) was plotted against PC1 in Figure 4. As it was shown in Figure 4, PC1 has an ability to discriminate true binders from decoys, as at the left side the density of true ligands is much higher. PC2 is not very informative in this regard.

## 4. Discussion

It was clear that MMPs are still interesting targets for pharmaceutical studies. On the other hand, scoring functions have different performance on different targets [3]. We used 11 scoring functions to predict the binding affinities for MMPs-inhibitor complexes. After that, the results were tested on a member of MMPs family (MMP-12). The F-Score, PoseScore, RankScore, DSX, and ChemScore showed the best performances among the 11 assessed scoring functions in scoring and ranking ligand-receptor binding taken from available MMPs crystal structures. In the next step we evaluated the scoring functions ability to find MMP-12 inhibitors (active compounds) among set of decoys. Our enrichment and ROC curve study further validated the results of predictivity study for DSX and ChemScore via analysis of MMP-12 virtual screening results. The PC1 component of PCA model consisting of three scoring functions (Autodock, DSX, and ChemScore) had the best performance in enrichment study.

The overall performance of scoring functions in prediction of experimental binding affinities of MMPs 3D structures in presence of inhibitors was not satisfying in comparison with those reported in some previous studies on other targets [6, 25, 30]. This could be due to the lack of restrictive selection criteria in our study. We did not apply restrictive selection criteria on our test set 3D structures, since it would dramatically decrease the statistical power of the analysis. Usually test sets for evaluation of docking/scoring functions include only X-ray crystallography structures with high resolutions. Moreover, the binary complexes are preferred. But our test set included complexes that are not fully suitable for docking/scoring studies. In addition, the binding affinity data were taken from different sources that could be the source of noises in analysis.

However, the scoring functions with top correlation coefficients (DSX and ChemScore) associated with the best ROC curve and EFs in rescoring virtual screening results of MMP-12. This was validated by the applicability of the predictivity power study results for MMPs. As the PCA potential for improving virtual screening results was demonstrated in previous reports [31], this approach was also evaluated in our study. PCA models obtained from different scoring functions were tested and the one which included Autodock, DSX, and ChemScore scoring functions had improved the ROC as well as EF of SP Glide virtual screening results.

The ultimate goal of this study was to determine which of the scoring functions or combinations of them would yield the best results in terms of enrichment when used against MMPs in a virtual screening study. Our study was retrospective and virtual screening was only performed in case of MMP-12. However, due to high similarity between active site structure and sequence among MMPs family, the similar results were expected for other members.

## Supplementary Material

For each PDB structure the detailed information (including experimental affinities) is presented. The complete correlation table of scoring functions (scores from all the 11 scoring functions as well as two consensus scorings) are shown.

## Figures and Tables

**Table 1:** Correlation coefficients (Pearson's and Spearman's correlation coefficients) for 11 individual and two consensus scoring functions with pAffinity.

	Pearson's correlation coefficient with pAffinity	Spearman's correlation coefficient with pAffinity
Consensus (rank-by-rank)	0.298	0.227
Consensus (rank-by-number)	−0.303	−0.211
AutoDock4.1	−0.049	0.019
ChemScore	−0.253	−0.216
D-Score	−0.090	−0.048
DSX	−0.368	−0.255
F-Score	−0.390	−0.391
G-Score	−0.178	−0.148
PoseScore	−0.321	−0.227
RankScore	−0.311	−0.285
PMF-Score	−0.148	−0.147
Vina	−0.078	−0.036
X-Score	−0.209	−0.109

**Table 2:** The scoring functions are ranked from the best (1) to the worst (5) according to the correlation with experimental data.

Based on Rp	F-Score^1^	DSX^2^	PoseScore^2^	RankScore^2^	ChemScore^3^	X-Score^4^	G-Score^4^	PMF-Score^4^	D-Score^5^	Vina^5^	AutoDock4.1^5^

Based on Rs	F-Score^1^	RankScore^2^	DSX^2^	PoseScore^2^	ChemScore^3^	G-Score^4^	PMF-Score^4^	X-Score^4^	D-Score^5^	Vina^5^	AutoDock4.1^5^

**Table 3:** The performance characteristics of scoring functions in discrimination of true binders after docking.

	Scoring method	AUC of ROC curve	EF20%	EF10%	EF2%	EF1%
Glide (HTS)	Glide	0.653348	2.142857	2.5	1.785714	3.571429
	F-Score	0.242776	0	0	0	0
	PMF-Score	0.501041	0.535714	0.714286	0	0
	G-Score	0.551684	1.428571	1.428571	5.357143	10.71429
	D-Score	0.515996	1.428571	1.785714	7.142857	7.142857
	ChemScore	0.648363	2.678571	3.571429	7.142857	14.28571
	X-Score	0.56054	1.25	1.785714	1.785714	3.571429
	DSX	0.632341	2.142857	2.857143	1.785714	0
	Autodock	0.646775	1.964286	2.142857	7.142857	10.71429
	Vina	0.560097	1.25	1.071429	0	0

Glide (SP)	Glide	0.730062	2.758621	3.448276	8.62069	13.7931
	F-Score	0.409975	0.172414	0.344828	0	0
	PMF-Score	0.496598	0.689655	0.689655	0	0
	G-Score	0.56524	1.724138	1.724138	1.724138	3.448276
	D-Score	0.549838	1.206897	1.724138	1.724138	0
	ChemScore	0.757174	2.758621	4.827586	12.72414	20.68966
	X-Score	0.605001	1.896552	1.724138	1.724138	3.448276
	DSX	0.683476	2.586207	3.793103	6.896552	10.34483
	Autodock	0.690234	1.896552	3.793103	12.06897	13.7931
	Vina	0.592455	1.551724	1.37931	1.724138	3.448276
	PC1	0.79963	3.448276	5.862069	18.96552	34.48276
