# Supplementary material for: Evaluation of 11 Scoring Functions Performance on Matrix Metalloproteinases
Source: Int J Med Chem. 2014 Dec 25;2014:162150. doi: 10.1155/2014/162150 (PMC4291136; doi:10.1155/2014/162150)
Supplement: Supplementary file 1 — For each PDB structure the detailed information (including experimental affinities) is presented. The complete correlation table of scoring functions (scores from all the 11 scoring functions as well as two consensus scorings) are shown. [file 162150.f1.doc]

Supplementary materials

Table 1. PDB codes and specifications of 3D structures used in predictivity test of scoring functions.

| PDB code | Type of MMP enzyme | PDB code for ligand(s) | Kd or Ki (nM) | IC50 (nM) | Resolution |
| --- | --- | --- | --- | --- | --- |
| 2TCL | MMP-1 | RO4 |  | 9-40 | 2.2 |
| 3AYK | MMP-1 | CGS | 33 | 2.5-96 | NMR |
| 966C | MMP-1 | RS2 | 23 |  | 1.90 |
| 1CIZ | MMP-3 | DPS | 36 |  | 1.64 |
| 1HFS | MMP-3 | L04 | 2 |  | 1.7 |
| 1CAQ | MMP-3 | DPS | 19 |  | 1.8 |
| 1USN | MMP-3 | IN9 | 18 |  | 1.8 |
| 1C3I | MMP-3 | TR1 | 1600 |  | 1.83 |
| 1B8Y | MMP-3 | IN7 | 14 |  | 2 |
| 1D7X | MMP-3 | SPC |  | 3-19 | 2 |
| 1G4K | MMP-3 | HQQ |  | 2000 | 2 |
| 2USN | MMP-3 | IN8 | 310 |  | 2.2 |
| 1B3D | MMP-3 | S27 | 24.4-9170 |  | 2.3 |
| 1BQO | MMP-3 | N25 | 18 | 18.4 | 2.3 |
| 1D8F | MMP-3 | SPI |  | 18 | 2.4 |
| 1D8M | MMP-3 | BBH |  | 3.1 | 2.44 |
| 1BIW | MMP-3 | S80 |  | 104 | 2.5 |
| 1D5J | MMP-3 | MM3 |  | 0.7 | 2.6 |
| 1SLN | MMP-3 | INH | 230 |  | NMR |
| 1UMT | MMP-3 | 0DS | 127 |  | NMR |
| 3USN | MMP-3 | ATT | 710 |  | NMR |
| 2Y6D | MMP-7 | TQJ |  | 79 | 1.6 |
| 2Y6C | MMP-7 | TQI |  | 10000-50000 | 1.7 |
| 1MMQ | MMP-7 | RRS | 3-30 |  | 1.9 |
| 1MMP | MMP-7 | RSS | 850 |  | 2.3 |
| 1MMR | MMP-7 | SRS | 4000 |  | 2.4 |
| 1ZS0 | MMP-8 | EIN MES | 700 | 810 | 1.56 |
| 3DPE | MMP-8 | AXB |  | 57 | 1.6 |
| 1BZS | MMP-8 | BSI EPE |  | 10 | 1.7 |
| 1ZVX | MMP-8 | FIN | 0.6 | 1.4 | 1.87 |
| 1JJ9 | MMP-8 | BBT |  | 1700 | 2 |
| 3DNG | MMP-8 | AXA |  | 7.4 | 2 |
| 1MNC | MMP-8 | PLH | 2 | 0.046 | 2.1 |
| 3DPF | MMP-8 | AXB |  | 57 | 2.1 |
| 1JH1 | MMP-8 | JST | 41000 |  | 2.7 |
| 1ZP5 | MMP-8 | 2NI |  | 1200000 | 1.80 |
| 2OVX | MMP-9 | 4MR |  | 2 | 2 |
| 2OVZ | MMP-9 | 5MR | 13 |  | 2 |
| 2OW0 | MMP-9 | 6MR |  | 201 | 2 |
| 2OW1 | MMP-9 | 7MR |  | 1 | 2.2 |
| 2OW2 | MMP-9 | 8MR |  | 6 | 2.9 |
| 1HV5 | MMP-11 | RXP CPS | 5 |  | 2.6 |
| 1Y93 | MMP-12 | HAE | 8000000 |  | 1.03 |
| 3F17 | MMP-12 | HS4 | 2.36 |  | 1.1 |
| 3F18 | MMP-12 | HS5 | 29.5-39.5 |  | 1.13 |
| 3F19 | MMP-12 | HS6 | 65.1 |  | 1.13 |
| 3F16 | MMP-12 | HS3 | 5.91 |  | 1.16 |
| 3F1A | MMP-12 | HS7 | 61.1 |  | 1.25 |
| 3LJG | MMP-12 | EEF HAE P6G | Ki 18.6 |  | 1.3 |
| 2HU6 | MMP-12 | 37A | 154000 | 149000 | 1.32 |
| 1RMZ | MMP-12 | NGH | Ki=4.3, Kd=10 |  | 1.34 |
| 3N2V | MMP-12 | JT5 | Ki 31 |  | 1.55 |
| 2WO9 | MMP-12 | 068 023 |  | 62 | 1.7 |
| 3EHX | MMP-12 | BDL | Ki=25 |  | 1.9 |
| 3EHY | MMP-12 | TBL | Ki=1400 |  | 1.9 |
| 3LIR | MMP-12 | EEC | Ki= 119 |  | 1.9 |
| 3F15 | MMP-12 | HS1 | 7.88 |  | 1.7 |
| 3LIK | MMP-12 | EEG HAE P6G | Ki=1.92 |  | 1.8 |
| 3LIL | MMP-12 | EEA HAE | Ki=8.3 |  | 1.8 |
| 3LK8 | MMP-12 | Z79 | 19.7 |  | 1.8 |
| 3LKA | MMP-12 | M4S HAE | 1500000 |  | 1.8 |
| 3NX7 | MMP-12 | NHK | Ki=7.6, Kd=7.88 |  | 1.8 |
| 3N2U | MMP-12 | D3X | Ki = 14.3 |  | 1.81 |
| 1ROS | MMP-12 | DEO |  | 1-1.7 | 2 |
| 2W0D | MMP-12 | CGS | 2 |  | 2 |
| 2WO8 | MMP-12 | 077 023 |  | 520 | 2 |
| 1UTT | MMP-12 | CP8 |  | 24000-100000 | 2.2 |
| 2WOA | MMP-12 | 576 023 |  | 1150 | 2.3 |
| 1UTZ | MMP-12 | PF3 |  | 14-1400 | 2.5 |
| 1JIZ | MMP-12 | CGS |  | 2 | 2.6 |
| 1YCM | MMP-12 | NGH | Ki=4.3, Kd=10 |  | NMR |
| 1Z3J | MMP-12 | NGH | Ki=4.3, Kd=10 |  | NMR |
| 2K2G | MMP-12 | DSV | 7.5 |  | NMR |
| 3ZXH | MMP-13 | E41 |  | 3 | 1.3 |
| 830C | MMP-13 | RS1 | Ki = 0.28-0.6 | 0.3-2 | 1.6 |
| 1XUC | MMP-13 | PB3 |  | 72 | 1.7 |
| 2YIG | MMP-13 | 5EL |  | 79.4 | 1.7 |
| 2OW9 | MMP-13 | SP6 HAE | Ki = 64 | 20-51 | 1.74 |
| 1XUD | MMP-13 | PB4 |  | 0.67-8 | 1.8 |
| 1XUR | MMP-13 | PB5 |  | 6600 | 1.85 |
| 3ELM | MMP-13 | 24F | Ki = 0.19 | 0.5 | 1.9 |
| 3KRY | MMP-13 | 3KR | 0.1 | 0.1 | 1.9 |
| 3O2X | MMP-13 | 3O2 EPE | Ki = 0.13 |  | 1.9 |
| 3I7G | MMP-13 | 732 |  | 430 | 1.95 |
| 3KEK | MMP-13 | 3EK | Ki = 4.4 |  | 1.97 |
| 1ZTQ | MMP-13 | 0.033 |  | 1.3-2.4 | 2 |
| 3LJZ | MMP-13 | LA3 EPE | Ki = 7.3 |  | 2 |
| 3KEC | MMP-13 | 3KE HAE |  | 58-130 | 2.05 |
| 4A7B | MMP-13 | 3W4 3W5 |  | 39.8 | 2.2 |
| 3I7I | MMP-13 | 518 |  | 620 | 2.21 |
| 1YOU | MMP-13 | PFD |  | 0.6-0.87 | 2.3 |
| 2OZR | MMP-13 | CG1 HAE |  | 0.67 | 2.3 |
| 3KEJ | MMP-13 | 3EJ |  | 75.9 | 2.3 |
| 2D1N | MMP-13 | FA4 | Ki =7 |  | 2.37 |
| 456C | MMP-13 | CBP | Ki = 0.17 |  | 2.4 |
| 2PJT | MMP-13 | 347 |  | 860 | 2.8 |
| 1FLS | MMP-13 | WAY |  | 8-33 | NMR |
| 1FM1 | MMP-13 | WAY |  | 8-33 | NMR |
| 1RM8 | MMP-16 | BAT | Ki = 1 |  | 1.8 |
| 2JSD | MMP-20 | NGH | 17 |  | NMR |

Table 2. Correlation matrix for 11 individual as well as two consensus scoring functions (Pearson’s correlation coefficient).

|  | pAffinity | Consensus (rank-by-rank) | Consensus (rank-by-number) | AutoDock4.1 | ChemScore | D-Score | DSX | F-Score | G-Score | PoseScore | RankScore | PMF-Score | Vina | X-Score |
| --- | --- | --- | --- | --- | --- | --- | --- | --- | --- | --- | --- | --- | --- | --- |
| pAffinity | 1.000 | 0.298 | -0.303 | -0.049 | -0.253 | -0.090 | -0.368 | -0.390 | -0.178 | -0.321 | -0.311 | -0.148 | -0.078 | -0.209 |
| Consensus (Rank.by.Rank) | 0.298 | 1.000 | -0.987 | -0.794 | -0.691 | -0.385 | -0.926 | -0.267 | -0.747 | -0.902 | -0.384 | -0.785 | -0.809 | -0.916 |
| Consensus (Rank by Score) | -0.303 | -0.987 | 1.000 | 0.801 | 0.690 | 0.445 | 0.928 | 0.297 | 0.750 | 0.889 | 0.368 | 0.795 | 0.805 | 0.918 |
| AutoDock4.1 | -0.049 | -0.794 | 0.801 | 1.000 | 0.760 | 0.353 | 0.697 | -0.041 | 0.721 | 0.605 | 0.005 | 0.570 | 0.794 | 0.828 |
| ChemScore | -0.253 | -0.691 | 0.690 | 0.760 | 1.000 | 0.222 | 0.697 | 0.004 | 0.296 | 0.542 | 0.071 | 0.412 | 0.671 | 0.782 |
| D-Score | -0.090 | -0.385 | 0.445 | 0.353 | 0.222 | 1.000 | 0.297 | 0.138 | 0.097 | 0.290 | -0.006 | 0.258 | 0.226 | 0.463 |
| DSX | -0.368 | -0.926 | 0.928 | 0.697 | 0.697 | 0.297 | 1.000 | 0.301 | 0.629 | 0.888 | 0.362 | 0.709 | 0.774 | 0.868 |
| F-Score | -0.390 | -0.267 | 0.297 | -0.041 | 0.004 | 0.138 | 0.301 | 1.000 | 0.088 | 0.283 | 0.367 | 0.227 | 0.169 | 0.052 |
| G-Score | -0.178 | -0.747 | 0.750 | 0.721 | 0.296 | 0.097 | 0.629 | -0.088 | 1.000 | 0.559 | 0.126 | 0.777 | 0.787 | 0.699 |
| PoseScore | -0.321 | -0.902 | 0.889 | 0.605 | 0.542 | 0.290 | 0.888 | -0.283 | 0.559 | 1.000 | 0.625 | 0.635 | 0.688 | 0.748 |
| RankScore | -0.311 | -0.384 | 0.368 | 0.005 | 0.071 | -0.006 | 0.362 | -0.367 | 0.126 | 0.625 | 1.000 | 0.118 | 0.057 | 0.037 |
| PMF-Score | -0.148 | -0.785 | 0.795 | 0.570 | 0.412 | 0.258 | 0.709 | -0.227 | 0.777 | 0.635 | 0.118 | 1.000 | 0.618 | 0.709 |
| Vina | -0.078 | -0.809 | 0.805 | 0.794 | 0.671 | 0.226 | 0.774 | 0.169 | 0.787 | 0.688 | 0.057 | 0.618 | 1.000 | 0.869 |
| X-Score | -0.209 | -0.916 | 0.918 | 0.828 | 0.782 | 0.463 | 0.868 | -0.052 | 0.699 | 0.748 | 0.037 | 0.709 | 0.869 | 1.000 |

Table 3. Correlation matrix for 11 individual as well as two consensus scoring functions (Spearman’s correlation coefficient).

|  | pAffinity | Consensus (rank-by-rank) | Consensus (rank-by-number) | AutoDock4.1 | ChemScore | D-Score | DSX | F-Score | G-Score | PoseScore | RankScore | PMF-Score | Vina | X-Score |
| --- | --- | --- | --- | --- | --- | --- | --- | --- | --- | --- | --- | --- | --- | --- |
| pAffinity | 1.000 | 0.227 | -0.211 | 0.019 | -0.216 | -0.048 | -0.255 | -0.391 | -0.148 | -0.227 | -0.285 | -0.147 | -0.036 | -0.109 |
| Consensus (Rank.by.Rank) | 0.227 | 1.000 | -0.989 | -0.793 | -0.743 | -0.424 | -0.946 | -0.229 | -0.840 | -0.903 | -0.351 | -0.852 | -0.849 | -0.921 |
| Consensus (Rank by Score) | -0.211 | -0.989 | 1.000 | 0.799 | 0.721 | 0.462 | 0.940 | 0.250 | 0.829 | 0.896 | 0.326 | 0.849 | 0.838 | 0.921 |
| AutoDock4.1 | 0.019 | -0.793 | 0.799 | 1.000 | 0.742 | 0.376 | 0.693 | -0.075 | 0.708 | 0.624 | -0.008 | 0.628 | 0.813 | 0.815 |
| ChemScore | -0.216 | -0.743 | 0.721 | 0.742 | 1.000 | 0.159 | 0.715 | -0.027 | 0.580 | 0.558 | 0.107 | 0.513 | 0.731 | 0.829 |
| D-Score | -0.048 | -0.424 | 0.462 | 0.376 | 0.159 | 1.000 | 0.358 | 0.112 | 0.250 | 0.321 | 0.021 | 0.358 | 0.219 | 0.418 |
| DSX | -0.255 | -0.946 | 0.940 | 0.693 | 0.715 | 0.358 | 1.000 | 0.231 | 0.733 | 0.880 | 0.323 | 0.814 | 0.812 | 0.875 |
| F-Score | -0.391 | -0.229 | 0.250 | -0.075 | -0.027 | 0.112 | 0.231 | 1.000 | 0.064 | 0.274 | 0.325 | 0.171 | -0.164 | 0.045 |
| G-Score | -0.148 | -0.840 | 0.829 | 0.708 | 0.580 | 0.250 | 0.733 | 0.064 | 1.000 | 0.658 | 0.073 | 0.839 | 0.788 | 0.820 |
| PoseScore | -0.227 | -0.903 | 0.896 | 0.624 | 0.558 | 0.321 | 0.880 | 0.274 | 0.658 | 1.000 | 0.576 | 0.732 | 0.745 | 0.744 |
| RankScore | -0.285 | -0.351 | 0.326 | -0.008 | 0.107 | 0.021 | 0.323 | 0.325 | 0.073 | 0.576 | 1.000 | 0.122 | 0.118 | 0.042 |
| PMF-Score | -0.147 | -0.852 | 0.849 | 0.628 | 0.513 | 0.358 | 0.814 | 0.171 | 0.839 | 0.732 | 0.122 | 1.000 | 0.685 | 0.762 |
| Vina | -0.036 | -0.849 | 0.838 | 0.813 | 0.731 | 0.219 | 0.812 | -0.164 | 0.788 | 0.745 | 0.118 | 0.685 | 1.000 | 0.883 |
| X-Score | -0.109 | -0.921 | 0.921 | 0.815 | 0.829 | 0.418 | 0.875 | 0.045 | 0.820 | 0.744 | 0.042 | 0.762 | 0.883 | 1.000 |
